# The Cutting Edge: The Role of mTOR Signaling in Laminopathies

**DOI:** 10.3390/ijms20040847

**Published:** 2019-02-15

**Authors:** Francesca Chiarini, Camilla Evangelisti, Vittoria Cenni, Antonietta Fazio, Francesca Paganelli, Alberto M. Martelli, Giovanna Lattanzi

**Affiliations:** 1 CNR National Research Council of Italy, Institute of Molecular Genetics, Unit of Bologna, 40136 Bologna, Italy; camilla.evangelisti@cnr.it (C.E.); vittoria.cenni@cnr.it (V.C.); 2 IRCCS Istituto Ortopedico Rizzoli, 40136 Bologna, Italy; 3 Department of Biomedical and Neuromotor Sciences, University of Bologna, 40126 Bologna, Italy; antonietta.fazio@studio.unibo.it (A.F.); francesca.paganell15@studio.unibo.it (F.P.); alberto.martelli@unibo.it (A.M.M.)

**Keywords:** mTOR, laminopathies, lamin A/C, Emery-Dreifuss muscular dystrophy (EDMD), Hutchinson-Gilford progeria syndrome (HGPS), autophagy, cellular signaling, metabolism, bone remodeling, ageing

## Abstract

The mechanistic target of rapamycin (mTOR) is a ubiquitous serine/threonine kinase that regulates anabolic and catabolic processes, in response to environmental inputs. The existence of mTOR in numerous cell compartments explains its specific ability to sense stress, execute growth signals, and regulate autophagy. mTOR signaling deregulation is closely related to aging and age-related disorders, among which progeroid laminopathies represent genetically characterized clinical entities with well-defined phenotypes. These diseases are caused by *LMNA* mutations and feature altered bone turnover, metabolic dysregulation, and mild to severe segmental progeria. Different *LMNA* mutations cause muscular, adipose tissue and nerve pathologies in the absence of major systemic involvement. This review explores recent advances on mTOR involvement in progeroid and tissue-specific laminopathies. Indeed, hyper-activation of protein kinase B (AKT)/mTOR signaling has been demonstrated in muscular laminopathies, and rescue of mTOR-regulated pathways increases lifespan in animal models of Emery-Dreifuss muscular dystrophy. Further, rapamycin, the best known mTOR inhibitor, has been used to elicit autophagy and degradation of mutated lamin A or progerin in progeroid cells. This review focuses on mTOR-dependent pathogenetic events identified in Emery-Dreifuss muscular dystrophy, *LMNA*-related cardiomyopathies, Hutchinson-Gilford Progeria, mandibuloacral dysplasia, and type 2 familial partial lipodystrophy. Pharmacological application of mTOR inhibitors in view of therapeutic strategies is also discussed.

## 1. Introduction

### 1.1. Lamin A and Lamin C

Lamin A and lamin C are the two major splicing products of the *LMNA* gene, which also encodes lamin A delta 10 and lamin C2 [1]. Lamin A/C forms polymers of around 3.5 nm in diameter [2], which are interconnected in a meshwork underneath the nuclear envelope. Further, lamin A and C are also found in the nucleoplasm, bound to chromatin-related proteins as LAP2 alpha [3] and BAF (barrier to autointegration factor) [4]. Lamin A is transcribed and translated as a precursor protein known as prelamin A, which is subjected to a complex post-translational processing yielding mature lamin A [1,5]. Prelamin A C-terminal CaaX box, which is typical of farnesylated proteins, undergoes farnesylation by farnesyl transferase, cleavage of the last three aminoacids by the zinc metallopeptidase STE24 (ZMPSTE24) and carboxymethylation by the isoprenylcysteine carboxyl methyltransferase (ICMT). Thereafter, further cleavage by ZMPSTE24 eliminates the last 15 aminoacids, thus producing a short peptide and mature lamin A [5]. Prelamin A and its processing pathway have been implicated in both physiological and pathogenetic mechanisms [6,7]. Thus, prelamin A plays a physiological role during myogenic differentiation in recruiting inner nuclear membrane proteins SUN1, SUN2 (Sad1 and UNC-84) [8], and Samp1 [9], required for proper myonuclear positioning. Moreover, prelamin A modulation during stress response is a physiological mechanism related to import of DNA repair factors [10] or activation of chromatin remodeling enzymes (Mattioli et al., in preparation). On the other hand, prelamin A accumulation in cells causes toxicity leading to cellular senescence [11] as well as organism ageing [1]. Mature lamin A and lamin C are usually considered as participating in the same cellular mechanisms, although some lamin C-specific pathways have recently emerged [12,13] and lamin C has been shown to form homodimers [14]. Lamin A/C has been implicated in nuclear structure, mechanosignaling, chromatin and genome organization, and cellular response to stress and cellular differentiation [1,5]. All these mechanisms are related to the occurrence of a high number of lamin post-translational modifications, such as phosphorylation, sumoylation, and acetylation, which influence lamin polymerization and lamin interactions with partner proteins [15]. Among the most relevant lamin partners are nuclear envelope proteins emerin, SUN1, SUN2, and nesprins, which form the so-called LINC complex, connecting the nucleus to the cytoskeleton [8,16]. Moreover, lamins bind and regulate translocation of some transcription factors, including SREBP1 [7], Oct-1 [17], Sp-1 [18], NRF2 [19], and mechanoresponsive myocardin-related transcription factor A (MRTFA) [20], and bind and stabilize pRb [21,22] in an Erk1/2-dependent mechanism [23]. Lamin A/C also influences chromatin organization through binding to chromatin-associated proteins such as BAF [4] and histone deacetylases [24]. Further, association of lamins with specific chromatin domains called lamina-associated domains (LADs) has been widely studied in recent years and shown to affect the transcriptional landscape in a cell-type-specific way [25,26].

A role for lamins in cellular signaling has been mostly described in models of muscle differentiation and in muscular laminopathies [15]. In particular, the phosphoinositide 3-kinase (PI3K)/AKT and Erk 1/2 pathway has been extensively investigated in mouse models of EDMD [27,28,29]. In the same context, a major player appears to be TGFβ 2 signaling. TGFβ 2 levels are increased both in EDMD patient serum [30] and in mouse models of muscular laminopathies [31] and in both cases TGFβ 2 elicits upregulation of fibrogenic molecules. TGFβ 2 signals through the mechanistic target of the rapamycin (mTOR) pathway, although different involvement of AKT, mTOR itself, or p70 ribosomal S6 kinase 1 (S6K1) occur depending on cell types [30]. Of note, it has been demonstrated that lamin A mutations causing MADA or other progeroid laminopathies are also able to trigger TGFβ 2 signaling with downstream effects on mTOR pathway and osteoclastogenic activity [32]. On the other hand, AKT is a lamin A and prelamin A kinase, which phosphorylates Serine 404 in the protein rod domain [33] and targets prelamin A to lysosomal degradation [34]. It is tempting to speculate that feedback mechanisms aimed at the maintenance of proper lamin A levels [34] could involve activation of mTOR under both normal and pathological conditions. This review is aimed at providing an overview of available data to stimulate a new interpretation and suggest new experimental approaches to the issue of an mTOR-lamin A relationship. 

### 1.2. Laminopathies

**Figure 1:**
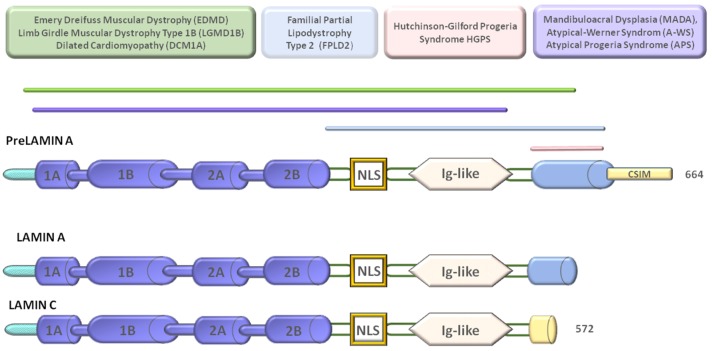
Diagram of prelamin A, lamin A, and lamin C structures with domains mutated in laminopathies. Laminopathies are grouped in the upper boxes referring to muscular laminopathies, lipodystrophy, Hutchinson-Gilford progeria syndrome (HGPS), and other progeroid laminopathies. Under physiological conditions and in muscular laminopathies, prelamin A is hardly detectable due to rapid maturation to lamin A. Most cases of laminopathies carry *LMNA* missense mutations. In progeroid laminopathies, prelamin A levels increase. In HGPS, truncated prelamin A (progerin) is accumulated due to a splicing defect. The pink bar spans the prelamin A domain missing in progerin. For bar colors, refer to the disease boxes.

Laminopathies are rare diseases caused by mutations in *LMNA* or other nuclear envelope genes or in genes structurally or functionally related to the nuclear envelope (Figure 1).

Most laminopathies are rare to very rare diseases and feature an autosomal dominant inheritance, although recessive inheritance can also occur, as in mandibuloacral dysplasia (MADA), Charcot–Marie tooth neuropathy (CMT2B1), and restrictive dermopathy (RD). Muscular laminopathies include muscular dystrophies characterized by joint contractures, muscle weakness and wasting, and cardiomyopathy—*LMNA*-linked congenital muscular dystrophy (L-CMD) and isolated cardiomyopathies with conduction defects [30,35]. Among muscular dystrophies, EDMD1 is linked to emerin mutations (*EMD* gene), EDMD2 and limb -girdle muscular dystrophy type 1B are caused by dominant lamin A/C mutations (*LMNA* gene), other forms of EDMD are caused by nesprin (*SYNE1* and *SYNE2* genes), FHL1, SUN1, or SUN2 mutations (Table 1) [1,30]. Cardiomyopathies occurring in the absence of muscular dystrophy have been so far mostly linked to *LMNA* mutations, although cases related to nesprin gene defects have also been reported, and an association of *LMNA* with modifier gene variants has been suggested [36,37,38]. 

Laminopathies featuring a lipodystrophy phenotype acquire partial lipodystrophy due to *LMNB2* gene mutations, type 2 familial partial lipodystrophy (FPLD2), type A and B mandiboloacral dysplasia (MADA and MADB) associated with mutations in *LMNA*, or the prelamin A endoprotease ZMPSTE24 gene, respectively. Lipodystrophy can be partial, causing loss of specific fat depots and fat accumulation in other districts, or generalized, as in MADB. MADA and MADB are also considered progeroid laminopathies since patient experience mildly accelerated ageing and bone and skin defects typical of aged individuals [1,58]. The latter symptoms are present with increased severity in HGPS, a premature aging syndrome with very early onset also causing cardiovascular disorders and premature death [58] (Table 1).

## 2. mTOR Complexes and Signaling Regulation

### 2.1. mTOR Complexes

**Figure 2:**
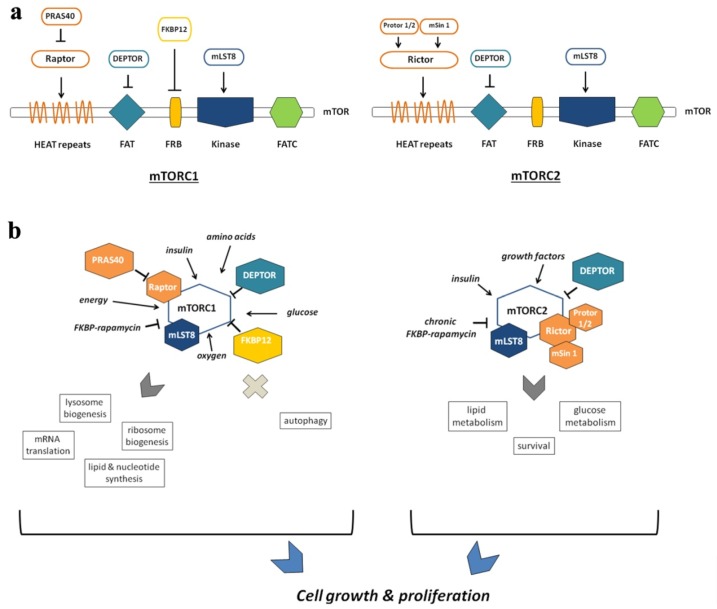
mTORC1 and mTORC2 domains and interactors. (**a**) Deptor, DEP domain-containing mTOR-interacting protein; FAT, FKBP/ATM/TRRAP; FATC, FRAP/ATM/TRRAP/Carboxy terminal; FKBP-12, FK506-binding protein-12; FRB, FKBP, rapamycin-binding; HEAT, Huntingtin/Elongation factor 3/A subunit of protein phosphatase-2A/ TOR1; mLST8, mammalian lethal with SEC13 protein 8; mSin1, mammalian stress-activated protein kinase interacting protein 1; mTOR, mechanistic target of rapamycin; mTORC1, mTOR complex 1; mTORC2: mTOR complex 2; PRAS40, proline-rich AKT substrate 1 40 kDa; Protor 1/2, protein observed with Rictor; Raptor, regulatory-associated protein of TOR; Rictor, rapamycin-insensitive companion of TOR. (**b**) mTORC1 and mTORC2 complexes and their role in cell growth and proliferation.

mTOR is a Ser/Thr kinase in the phosphoinositide kinase-related family of protein kinases (PIKK) [60]. Other members of the PIKK family include ataxia telangiectasia mutated (ATM), ataxia telangiectasia gene product- and RAD3-related (ATR), human suppressor of morphogenesis in genitalia-1 (hSMG-1), and the catalytic subunit of DNA-dependent protein kinase (DNA-PK) (Figure 2) [61].

mTOR works as a crucial integrator of growth factor-activated and nutrient sensing pathways to coordinate several cellular functions, and linking nutrient availability with metabolic control [62]. mTOR is the catalytic subunit of two functionally and structurally distinct protein complexes known as mTORC1 and mTORC2 (Figure 2a,b) [60].

#### 2.1.1. mTORC1

mTORC1 is composed of mTOR, RAPTOR (regulatory-associated protein of mTOR), which is important for the subcellular localization of mTORC1, mLST8 (mammalian lethal with Sec13 protein 8), PRAS40 (proline rich AKT substrate 40 kDa), and DEPTOR (DEP-domain containing mTOR interacting protein), the latter two having an inhibitory function on mTORC1 (Figure 2).

**Figure 3:**
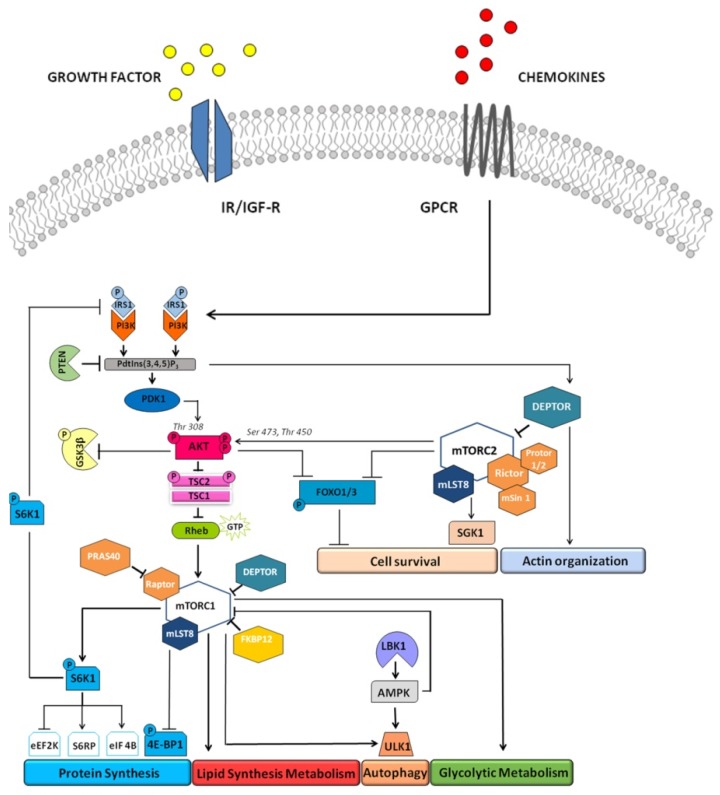
mTOR signaling network. The serine/threonine kinase mTOR is found in two multiprotein complexes: mTORC1 is composed of RAPTOR, PRAS40, mLST8, and DEPTOR, which has an inhibitory function on mTORC1. The mTORC1 is activated by growth factors, chemokines, nutrients (glucose, amino acids), and the cell energy status (i.e., a high ATP/AMP ratio). mTORC1 stimulation is activated by growth factors through the phosphoinositide 3-kinase (PI3K)-AKT signaling pathway. AKT phosphorylates tuberous sclerosis complex 2 (TSC2 or hamartin) at multiple sites. TSC2 is a GTPase-activating protein (GAP) that associates with tuberous sclerosis 1 (TSC1 or tuberin) for inactivating the small G protein Rheb (Ras homolog enriched in brain). Once AKT phosphorylates TSC2, the GAP activity of the TSC1/TSC2 complex is repressed, allowing Rheb to accumulate in a GTP-bound state. Therefore, Rheb-GTP upregulates mTORC1 protein kinase activity. AKT also phosphorylates PRAS40 (at Thr246), which dissociates from mTORC1 in response to growth factors, or glucose and nutrients, thereby releasing the inhibitory function of PRAS40 on mTORC1. mTORC1 activates S6K1 through phosphorylation, and S6K1 in turn phosphorylates or binds proteins such as eukaryotic elongation factor 2 kinase (eEF2K), which targets eEF2 and regulates the elongation step of protein translation, ribosomal protein S6 (S6RP), and eukaryotic initiation factor 4B (eIF4B), ultimately promoting translation initiation and elongation. mTORC1 also phosphorylayes and inactivates the translation inhibitor 4E-BP1, which has a role in inhibiting cap-dependent translation through the binding of the translation initiation factor eIF4E. mTORC1 is a repressor of autophagy, through phosphorylation of Unc-51 like autophagy activating kinase 1 (ULK1), and positively controls lipid synthesis and glycolytic metabolism.

Multiple inputs converge on mTORC1: growth factors, chemokines, nutrients (glucose, amino acids), and the cell energy status (i.e., a high ATP/AMP ratio) [63]. Growth factors and cytokines stimulate mTORC1 mostly through the PI3K/AKT signaling pathway. However, growth factors and chemokines signal to mTORC1 also occur through the Ras/Raf/MEK/ Erk 1/2 network. Moreover, recent studies have shown that mTORC1 and mTORC2 also respond to inputs via the Wnt and liver kinase 1 (LKB1)/AMP-activated protein kinase (AMPK) signaling pathways (Figure 3) [62]. 

Upon activation, mTORC1 regulates ribosomal and lysosomal biogenesis, cap-dependent translation, autophagy, mitochondrial biogenesis, lipid synthesis, and thermogenesis, acting through direct phosphorylation of many substrates. mTORC1 can also sustain nucleotide biogenesis promoting the expression of genes involved in the pentose phosphate pathway and pirimidine biosynthesis (Figure 3) [64]. 

It has been demonstrated that reducing mTOR activity increases autophagic flux, reducing reactive oxygen species within the cell and increasing replicative lifespan. At the same time, it has been established that impaired mitochondrial function is associated with aging and age-related diseases, in which decreased mitochondrial function can have a significant impact on ATP production, maintenance of NAD/NADH ratios, and reactive oxygen species (ROS) production [65]. In the case of laminopathies, defective mitochondrial activity has been documented in FPLD2 [66], models of DCM [67], and HGPS cells [68].

Activation of mTORC1 relieves the inhibitory effect of 4E-BP1 on eIF4E and stimulates the translation of nuclear-encoded mRNAs of mitochondrial proteins, including the mitochondrial transcription factor A (TFAM) and mitochondrial ribosomal proteins and components of complex I and V. In addition, mTORC1 regulates mitochondrial function by modulating transcription of mitochondrial nuclear-encoded genes via Yin Yang 1 (YY1) and peroxisome proliferator-activated receptor-gamma coactivator 1 α (PGC-1α) [69]. Hence, it is likely that mTORC1 stimulates mitochondrial function by orchestrating translation and transcription of distinct mitochondria-related genes [70].

It has been demonstrated in models of senescence and Hutchinson–Gilford progeria that senescence is accompanied by elevated glycolysis and increased oxidative phosphorylation, which are both reduced by rapamycin or rapalogs [68]. 

Concerning protein synthesis, mTOR is able to control the balance between anabolism and catabolism in response to environmental cues [71] and to act through phosphorylation of the effectors of the protein synthesis machinery, including S6K1 and the translation inhibitor, eukaryotic translation initiation factor 4E-binding protein 1 (4E-BP1) [72,73]. S6K1 is phosphorylated by mTORC1 in Th389, enabling its complete phosphorylation and activation by PDK1. S6K1 phosphorylates ribosomal protein S6 (RPS6), leading to active translation of mRNAs involved in ribosome biogenesis. S6K1 has also several substrates, including insulin receptor substrate-1 (IRS1), which is upstream of mTORC1. Importantly, IRS1 phosphorylation by S6K1 is fundamental for its proteasomal degradation and impairs growth factors (insulin, insulin-like growth factor-1) signaling downstream of receptor tyrosine kinases (RTKs) [60]. The consequence is a negative feedback loop, in which PI3K/AKT axis is inhibited and mTORC1 activity is regulated. In addition, also Grb10 acts as a negative regulator of mTORC1 through phosphorylation-dependent feedback mechanisms. Regarding 4E-BP1, its phosphorylation by mTORC1 triggers its dissociation to the cap-binding protein eukaryotic initiation factor 4E (eIF4E), enabling it to constitute the eIF4F complex, necessary for the initiation of cap-dependent mRNA translation (Figure 3) [60,72].

Further, mTORC1 acts increasing the glycolytic flux, promoting a shift in glucose metabolism, by activating the transcription and the translation of hypoxia inducible factor 1α (HIF1α), a positive regulator of many glycolytic genes, and facilitating cell growth [60].

mTORC1 also controls de novo lipid synthesis, necessary for proliferating cells to generate membranes, acting mainly through the sterol regulatory element-binding proteins 1 (SREBP1), which are transcription factors of lipogenic genes, as well as through peroxisome proliferator-activated receptor gamma (PPARγ), the master regulator of adipogenesis [60]. 

In addition to various anabolic roles, mTORC1 is a negative regulator of autophagic processes, required to eliminate damaged organelles and recycle molecules as well as for cellular adaptation to nutrient starvation [74]. When mTORC1 is inhibited, autophagosomes sequester cytoplasmic components and then fuse with lysosomes, leading to degradation of cell components and recycling of cellular building blocks. 

mTORC1 directly phosphorylates and suppresses ULK1/Atg13/FIP200 (unc-51-like kinase 1/mammalian autophagy-related gene 13/focal adhesion kinase family-interacting protein of 200 kDa), a kinase complex which is required to drive autophagosome formation [75]. mTORC2 is composed of RICTOR, SIN1, mLST8, PROTOR, and DEPTOR. It regulates cell survival through serum and glucocorticoid activated kinase 1 (SGK1) and AKT. mTORC2 phosphorylates AKT on Ser473, priming AKT for further phosphorylation by PDK1 at the Thr308 residue. Loss of phosphorylation at Ser 473 site, however, only affects some of AKT substrates, such as FOXO transcription factors, but not TSC2, in response to growth factor signaling. mTORC2 associates with actively translating ribosome to co-translationally phosphorylate AKT (at Thr450), which prevents ubiquitinylation and degradation of AKT. mTORC2 is involved in the spatial control of cell growth via cytoskeleton regulation. Arrows indicate activating events, while perpendicular lines indicate inhibitory events. GPCR: G protein-coupled receptor; IGF-R, insulin-like growth factor receptor; IR: insulin receptor.

On the contrary, during nutrient deprivation, lysosomal mTORC1 is inactivated, partially due to lack of amino acids. Under energy starvation conditions, an increase in AMP levels stimulates the activity of AMPK, and inhibits mTORC1. It is observed that the relative activity of mTORC1 and AMPK in different contexts may determinate the level of autophagy induction. AMPK, which is activated by LKB1 under metabolic stress conditions, phosphorylates ULK1 at multiple sites, thus upregulating ULK1 activity, and activates tuberous sclerosis complex 2 (TSC2), which is an indirect inhibitor of mTORC1 activity. Following mTORC1 inhibition, derepressed ULK1, by binding to ATG14L (ATG—autophagy related-14 Like), is recruited to a molecular platform composed of vacuolar protein sorting 34 (Vps34) and beclin-1. This leads to the phosphorylation of beclin-1 on Ser 14, and activation of Vps34, which has an important role in regulating membrane trafficking and autophagy, releasing PI3-phosphate at the nascent autophagosome [76]. In this condition, microtubule-associated protein 1 light chain 3 (LC3), a structural protein found in the cytoplasm in its precursor form (LC3I), is cleaved, coupled to phosphatidylethanolamine and converted into its active autophagosomal, membrane-bound form, LC3II [76]. 

Protein turnover is controlled by the ubiquitin proteasome system (UPS), through which proteins are targeted for degradation by the proteasome after ubiquitination. mTORC1 was recently found involved in the control of proteasome-dependent proteolysis. Acute mTORC1 inhibition seems to increase proteasome-dependent proteolysis likely to restore aminoacid pools. Prolonged hyperactivation of mTORC1 signaling increases proteasome activity through elevated expression of proteasome subunits downstream of Nrf1, as a compensatory mechanism to balance the increase in protein turnover and protein synthesis [77]. 

#### 2.1.2. mTORC2

mTORC2 is composed of RICTOR (rapamycin-insensible companion of mTOR), SIN1 (stress activated protein kinase-interacting 1), PROTOR1/2 (protein observed with Rictor), mLST8, and DEPTOR (Figure 2b) [60]. Unlike mTORC1, mTORC2 is not sensitive to nutrients, but it responds to growth factors via the PI3K signaling pathway. In fact, the most relevant role of mTORC2 is AKT phosphorylation in Ser473 and its activation, which is required for the phosphorylation of Forkhead box O 1/3a (FoxO1/3a) transcription factors. This phosphorylation allows AKT to promote cell growth and survival via PI3K/AKT signaling. AKT also phosphorylates and inhibits GSK3beta and the mTORC1 inhibitor TSC2. Thus, mTORC2 acts as an effector of insulin/PI3K signaling. The mTORC2 protein mSin1 contains a PH domain, essential for the insulin-dependent regulation of mTORC2 activity. mSin1 inhibits the catalytic activity of mTORC2 in the absence of insulin, and this autoinhibition is relieved upon binding to PIP3 generated by the PI3K at the plasma membrane (Figure 3). Localization at the plasma membrane is a key aspect of mTORC2 regulation. mSin1 can also be phosphorylated by AKT, with a positive feedback loop, while the partial activation of AKT promotes mTORC2 activation, which causes in turn AKT phosphorylation (and activation) at Ser473 [78]. 

mTORC2 controls cell survival/metabolism also through serum and glucocorticoid activated kinase 1 (SGK1), and it is involved in the spatial control of cell growth via cytoskeleton remodeling, through actin fibers, paxilin, RhoA, Rac1, and protein kinase C (PKC) family phosphorylation, all of which regulate several aspects of cytoskeleton remodeling and cell migration [60].

mTORC2 signaling is also regulated by mTORC1, due to the negative feedback loop between insulin/PI3K and mTORC1. In fact, mTORC1 can phosphorylate and activate Grb10, a negative regulator of insulin/IGF1receptor signaling, upstream of AKT and mTORC2, while S6K1 also suppresses mTORC2 activation through the phosphorylation dependent degradation of IRS1, as has been mentioned [78]. 

## 3. mTOR Signaling in Physiological Conditions

### 3.1. mTOR Signaling in Muscular Tissue

As in all the other tissues of the organism, in normal skeletal and cardiac muscle, the mTOR pathway plays a pivotal role in cell growth, proliferation, and survival. In particular, in skeletal muscle cells, mTOR controls both the anabolic and catabolic signaling resulting in the modulation of muscle hypertrophy and muscle wasting [79]. mTOR functions as a positive regulator of muscle hypertrophy, being downregulated by muscle atrophy-inducing signals, such as myostatin [80] and glucocorticoids [81] as well as by sarcopenia, an age-related decline in muscle mass due to a reduction of circulatory IGF1 levels. On the other hand, mTOR is strongly activated by anabolic stimuli such as muscle contraction, insulin, IGF1, and nutrients, and triggers an increase of protein synthesis and as such of muscle hypertrophy. In the cardiovascular system, mTOR activity is involved in the regulation of embryonic cardiovascular development and in the control of vital cellular processes necessary for postnatal growth and maintenance of cardiac function. Ablation of cardiac mTOR in murine models is in fact associated with a high rate of embryonic lethality [82], and cardiac disruption of mTORC1 activity is associated with cardiac dilation, dysfunction, apoptosis, mitochondrial and metabolic derangements, heart failure, and ultimately mortality in the postnatal stage [83]. In addition, complete genetic disruption of mTORC1 impairs the ability of the heart to respond to pressure overload and to undergo compensatory hypertrophy, resulting in the development of dilated cardiomyopathy [83]. On the other hand, mTOR inhibition triggers autophagy, protects cardiomyocytes during energy deprivation [84], extends lifespan, and reduces cardiac hyperthrophy in aged mouse models [85]. All these data suggest a complex and multifaceted role of mTOR signaling in the heart [86].

### 3.2. mTOR Signaling in Adipose Tissue

mTOR signaling plays a critical role in the regulation of adipogenesis, lipid metabolism, thermogenesis, and adipokine synthesis/secretion. However, a complex picture emerges from literature data, which suggests a condition highly dependent on environmental stimuli, adipose tissue depot, developmental stage, and the type of adipocyte precursors involved. White and brown adipose tissue have diverse morphology and functions [87]. Whereas white adipocytes are formed by large lipid droplets deputed to store energy in the form of triglycerides, brown adipocytes present small droplets and convert lipid-derived chemical energy into heat for thermogenesis [87]. mTORC1 is indispensable for adipose tissue homeostasis, as indicated by the occurrence of lipodystrophy, defects in dietary lipid intake and metabolic disorders in mice lacking mTORC1 in all mature adipocytes [88]. However, knocking out all adipocyte AKT activity by simultaneously deleting AKT1 and AKT2 in adipocytes, elicits an even more severe lipodystrophy phenotype, showing that AKT also regulates mTOR-independent adipocyte activities and that mTORC1-AKT interplay contributes to adipose tissue maintenance [88]. On the other hand, mTOR inhibition leading to the activation of autophagy contributes to white adipose tissue formation. Consistently, partial knockdown of mTOR increases adipogenesis, although complete inhibition of activity as well as inactivation of pS6K1 impairs adipocyte differentiation [89]. Several genes including PPARγ, a master regulator of adipogenesis, are upregulated by mTOR inhibition [89]. However, the best-known effect of mTOR downregulation, i.e., autophagy induction, plays a major role. In fact, autophagy is required for white adipogenesis [90], while inhibition of autophagy has been shown to convert white adipocytes into brown-like cells called brite or beige adipocytes [91]. Autophagy triggers degradation of PPARγ 2 proteases, thus contributing to PPARγ 2 increase. Further, autophagy promotes vesicle fusion, thus triggering a formation of large lipid droplets in white adipocytes [92]. Nevertheless, a recent paper shows that the thyroid hormone triiodothyronine (T3) stimulates autophagy in brown adipose tissue by reducing mTOR activity, and this elicits mitophagy and a more efficient mitochondrial respiratory chain [93]. 

### 3.3. mTOR in Bone Turnover

Mammalian bones are formed through two different mechanisms, endochondral or intramembranous ossification. During intramembranous ossification, mesenchymal progenitors directly differentiate into osteoblasts, while, in endochondral bone formation, a first stage of cartilage production is followed by remodeling of hypertrophic cartilage by osteoclasts and the production of bone matrix by osteoblasts [94]. mTORC1 has been studied in models of cartilage formation and chondrocyte differentiation, and diverse mechanisms have been proposed and partially validated by experimental evidence [95]. Further, mTORC1 and mTORC2 have been implicated in regulating osteoblast differentiation and function. Bone marrow stromal cells lacking Rictor gene exhibited reduced osteogenic potential and an increased tendency to undergo adipogenic differentiation, suggesting that mTOR signaling may regulate cellular fate, thus affecting bone homeostasis [95]. mTORC1 is required for the transition of preosteoblasts to mature osteoblasts and both mTORC1 and mTORC2 dysregulation have been linked to osteoarthritis and osteoporosis [95]. Moreover, mTOR activity affects osteoclasts and bone resorption, although effectors are not fully elucidated [95]. 

## 4. EDMD, DCM and Other Muscular Laminopathies

### 4.1. Muscular Laminopathies

*LMNA*-associated muscular laminopathies include EDMD2 and EDMD3 [96], DCM [97,98], LGMD1B [99], *LMNA*-related congenital muscular dystrophy (L-CMD) [100], and “heart-hand” syndrome (HHS) [101]. 

To date, there are multiple hypotheses regarding the onset of muscular dystrophies due to genetic mutations on *LMNA* or genes encoding for lamin A/C-associated proteins. The first one is the “structural hypothesis,” which suggests that a loss of structural integrity of the nuclear lamina leads to nuclear structural weakness, which ultimately results in a decrease in the ability of the nucleus to resist to the high mechanical strain typical of skeletal muscles [102]. This theory is validated by the fact that lamin A/C interacts with structural proteins including emerin SUN1 [8], nesprins, LAP2α [103], and Ankrd2 [104]. The interaction of lamin A/C with SUN proteins and nesprins is particularly relevant for nuclear positioning in muscle [8], and mutations in lamins affect LINC complex-lamin A/C interplay, thus leading to myonuclear clustering, a further determinant of *LMNA* pathogenetic mechanisms [8,9]. The “gene-expression” hypothesis suggests that some lamin A/C mutations may alter gene expression during muscle differentiation. Supporting this hypothesis, A-type lamins and some of their binding partners (e.g., LAP2 or emerin) interact with muscle specific transcription factors such as MyoD [105]. Moreover, myoblasts with altered lamin A/C or emerin are characterized by low levels of proteins involved in cell cycle regulation and muscle differentiation, such as MyoD, desmin, pRb, and M-cadherin [106]. Furthermore, the microRNA profile in skeletal muscles of patients affected by muscular laminopathies has revealed a significant alteration of proteins involved in muscle repair pathways, such as MAPK, TGF-β, and Wnt, as well as in differentiation and regenerative processes [107]. These pathogenetic hypotheses are not mutually exclusive and most likely concur to the onset of the pathology. 

#### mTOR Signaling in Muscular Laminopathies 

Several studies indicate that the molecular pathway ruled by mTOR is deeply affected in muscular laminopathies. As a consequence of the sustained mechanical strain to which they are constantly subjected, cardiac and skeletal muscle cells feature a particularly high amount of waste material, including mitochondrial-derived ROS (reactive oxygen species) and toxic molecules [108]. In normal conditions, ROSs block mTORC1 activity through the already described AMPK/mTOR pathway, triggering the activation of autophagy, that results in a prompt degradation of toxic molecules and dysfunctional organelles (for a review of autophagy regulation by ROSs, please read Filomeni et al. [109]. 

In muscle cells from laminopathic patients, there is an overall increase in the amount of ROS, due to both the reduction of nuclear plasticity [104] and the loss of the properties of “ROS acceptor” of the altered nuclear lamina unable to neutralize cellular ROSs [110]. In spite of this, the significant drop of autophagic activity has been described in models of muscular laminopathies. In 2012, the group of B. Kennedy demonstrated that, in the heart of *Lmna*^−/−^ mice suffering of muscular dystrophy and cardiomyopathy, the increased levels of LC3-BII, Atg7, and beclin 1 proteins, generally indicating an ongoing autophagic pathway, were not followed by a decrease in p62/SQSTM1 [111], which is a marker of an active autophagic flux. Almost simultaneously, the group of H. Worman demonstrated that cardiac cells from a mouse model carrying the p.H222P lamin A/C mutation causing EDMD2 in humans exhibited defective autophagy in response to starvation [97,112]. Interestingly, in this study the authors also found a clear correlation between the expression level of mutated lamin A/C and the hyperactivation of both AKT and Erk 1/2 [97,112], which results in the activation of mTORC1. Finally, the confirmation that the mTORC1/autophagy axis plays a central role in the pathophysiology of cardio-muscular laminopathies, came from the evidences that rapamycin or its analog temsirolimus improve cardiac and muscle functions, and extend the lifespan in both laminopathic mouse models [97,111]. In serum from patients affected by muscular laminopathies, a wide study conducted by the Italian Network for Laminopathies demonstrated a significant increase in TGFβ 2 levels [30]. This was associated with hyperactivation of mTOR in cultured myoblasts and AKT and pS6K1 in fibroblasts [30]. Intriguingly, in both cell types, neutralization by a TGFβ 2 antibody rescued mTOR or AKT hyperactivation, thus showing a major role of TGFβ 2 in the signaling pathway in EDMD2 [30]. Downstream events in that experimental context reduced myoblast differentiation and fibrogenic conversion of myoblasts ad tenocytes [30], the latter being a poorly investigated cell type with potential involvement in joint contractures typical of EDMD. The relevance of TGFβ 2 signaling in pathogenetic events, mostly leading to fibrogenic conversion of myoblasts, has also shown in the H222P/H222P *Lmna* mouse model of EDMD2 [31]. 

The group of B. Kennedy also wondered if mTOR was involved in the activation of pathways promoting metabolic response via phosphorylation of S6K1 and 4E-BP1 [113]. The authors reported that heterozygosity for S6K1 (S6K1^+/−^) extended lifespan of *Lmna*^−/−^ mice exactly as rapamycin treatment did. Intriguingly, life extension of *Lmna*^−/−^ S6K1^+/−^ mice was not due to improvement in cardiac function (as seen for rapamycin treatment) or to the rescue of metabolic alterations, but to the amelioration of skeletal muscle deficits. In contrast, whole-body overexpression of 4E-BP1 shortened the survival of *Lmna*^−/−^ mice, likely by accelerating lipolysis, pointing to the conclusion that rapamycin possibly extends survival of *Lmna*^−/−^ mice through the mTORC1-S6K1 branch, but not the mTORC1/4E-BP1 one of the mTOR signaling pathway [113]. 

Altered autophagic activity has been also observed in hearts of transgenic *Drosophila* expressing mutated LamC (the genetic counterpart of human *LMNA*) and affected by cardiomyopathy [114]. In this model, mutant Lamin C accumulated in the cytosol, resulting in upregulation and accumulation of p62/SQSTM1. These events caused inactivation of AMPK, activation of mTOR, and ultimately inhibition of autophagy [114]. Interestingly, similar evidence had been previously obtained in heart and muscle from other transgenic *Drosophila* models carrying other EDMD2-causative mutations in LamC [115]. In support to mTOR signaling pathway alteration in laminopathic muscle cells, cardiac cells from transgenic flies also featured metabolic alterations, including an age-dependent increase in size of fat bodies and enhanced triglyceride amount [114]. Overexpression of *Atg1* (a kinase promoting autophagy) suppressed cardiac defects associated with mutant LamC and restored cardiac function and lifespan. At the molecular level, the reactivation of autophagy induced by *Atg1* overexpression elicited clearance of cytoplasmic LamC aggregates, reduction of p62 accumulation, reactivation of AMPK pathway, and ultimately restoration of mTOR activity [114]. 

Finally, in support of the involvement of mTORC1 signaling in the pathophysiology of muscular laminopathies, it is important to emphasize some results obtained from muscular models with a phenotype very similar to the dystrophic one, that is aged non-pathological muscle. Aged muscle cells have in fact several traits in common with dystrophic cells, such as reduced regenerative and differentiative potential and high levels of basal ROSs. In 2015, the group of E. Volpi reported that, despite a basal level of hyperphosphorylated mTORC1, aged muscle cells presented a poor protein synthesis, suggesting that, in these model, mTORC1 phosphorylation was not sufficient per se to activate protein synthesis pathway as usually did [116]. In another study, performed in aged muscles of a TSC1 KO mice, it was observed that chronic mTORC1 activation, obtained by the lack of TSC1, did not lead to muscle hypertrophy, mainly because of the inability to induce autophagy [117]. Similar to that reported regarding cardio-laminopathic mice [97,111], in aged and stressed heart, mTORC1 inhibition, obtained by the use of selective mTOR inhibitors, resulted in cardioprotection and extended lifespan [118], reducing cardiac hypertrophy and improving cardiac function in the presence of pressure overload [119]. 

**Figure 4:**
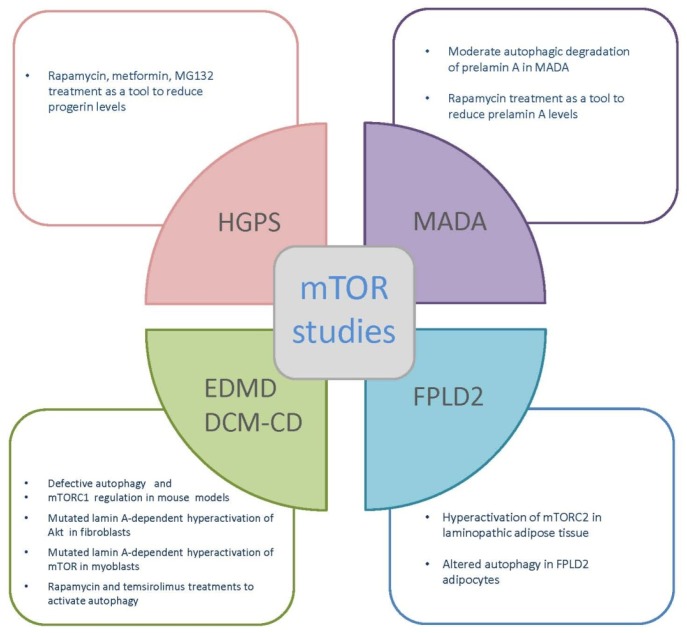
mTOR studies in laminopathies. Schematic representation of mTOR studies in EDMD, HGPS, MADA, and FPLD2. For EDMD see refs: [97,112]; for HGPS see refs: [120]; for MADA see refs: [5]; for FPLD2 see refs: [121] (Pellegrini et al., in preparation).

These findings independently obtained from different models of laminopathic and aged muscles perfectly support each other, and corroborate the relevance of altered autophagic processes in muscular laminopathies (Figure 4).

## 5. FPLD2

### 5.1. Laminopathic Lipodystrophies

Monogenic causes of lipodystrophies mostly converge in primary alterations of the adipose tissue, such as impaired defects in the formation, maintenance, and regulation of the adipocyte lipid droplets, leading to a loss of fat in specific district or in the whole body and secondary metabolic dysfunction [53]. As said above, mutations in lamin A/C, lamin B2, or ZMPSTE24 gene or alterations of prelamin A maturation are the cause of laminopathies featuring lipodystrophy. 

From a clinical point of view, FPLD2 and MADA feature type A lipodystrophy, i.e., a loss of fat from the limbs and trunk and accumulation in the neck, while MADB presents with a generalized loss of adipose tissue (type B lipodystrophy) [122]. Metabolic alterations such as insulin resistance, diabetes, dyslipidemia, and nonalcoholic fatty liver diseases are found in laminopathic lipodystrophies with some variability among individuals [51]. The onset of lipodystrophy is at puberty, while up to that age most mutation carriers appear unaffected [122]. Of note, fat loss is much more evident in females and some male patients remain asymptomatic for several years [122]. A main clinical phenotype in MADA and MADB is accelerated ageing with onset in the second decade [1].

#### FPLD2

FPLD2 is typical partial lipodystrophy caused by mutations in the *LMNA* gene. More than 85% of FPLD2 mutations affect arginine 482 (p.Arg482Trp, p.Arg482Gln, p.Arg482Leu), located in exon 8, which encodes the globular portion of the protein. Different *LMNA* missense mutations have been reported in patients with FPLD2, most of them occurring in lamin A/C C-terminal domains [123,124].

FPLD2 is characterized by a loss of fat from the limbs, buttocks, and trunk, with cushingoid appearance, due to fat accumulation in the neck, face, and axillary regions [51,53,122]. In addition, patients may present muscular hypertrophy. FPLD2 patients also show early-onset atherosclerosis leading in some cases to cardiovascular pathologies and premature coronary heart disease, peripheral arteritis, and stroke [125].

Adipose tissue endocrine functions are also affected, with decrease in adiponectin- and leptin-circulating levels, leading to a worse prognosis in female patients, thus indicating that steroids could be involved in lipodystrophic phenotypes, resulting from *LMNA* mutations [124]. 

At the molecular level, FPLD2, MADA, and MADB are also characterized by accumulation of prelamin A at the nuclear periphery [66,126]. In FPLD2 and MADA, prelamin A accumulation is associated with recruitment of BAF to the nuclear periphery [127] and interferes with import and transactivation activity of the adipocyte transcription factor SREBP1 [7]. With a similar mechanism, accumulation of prelamin A by treatment of cells with anti-retroviral protease inhibitors, drugs that caused a lipodystrophy phenotype in patients subjected to therapy, has been shown to affect Sp1 import in nuclei of mesenchymal stem cells and Sp1-dependent regulation of genes related to lipid metabolism [18]. 

In FPLD2, it was observed that lamin A connection with chromatin at the nuclear periphery and in the nuclear interior could be affected and associated with tridimensional rearrangements of chromatin [128]. 3D genome models of fibroblasts from FPLD2 patients have shown a repositioning in the nuclear center of the T/Brachyury gene, a key regulator of mesodermal differentiation, an event favoring gene transcription [129]. 

To address FPLD2 pathogenesis, the particular clinical condition must be considered. Patients lose subcutaneous fat, while they accumulate adipose tissue in the neck and in some instances in visceral depots [122]. In a study aimed at evaluating different fat districts, it was shown that prelamin A accumulation in lipoatrophic fat is associated with altered expression of cyclin D3, pRB, and PPARγ genes, involved in adipocyte differentiation and proliferation [130]. On the other hand, fibrosis, altered expression of adipogenic factors, a mitochondrion number increase, and enhanced levels of uncoupling protein 1 (UCP1, a marker of brown adipocytes) have been reported in the neck adipose tissue from FPLD2 patients, suggesting that the hypertrophic adipose tissue found in that particular district is of brown origin [126,130]. It is plausible that *LMNA* defects might affect a group of adipogenic genes or differentiation mechanisms depending on the type of adipogenic precursors [5]. Our recent work has demonstrated that impaired autophagy due to hyperphosphorylation of pS6K1 contributes to downregulation of white adipose tissue genes in cells from FPLD2 patients, while aberrant activation of autophagy in brown preadipocytes from the neck of FPLD2 patients contributes to direct cell differentiation towards a white adipocyte phenotype (Pellegrini et al., in preparation). As a consequence of impaired autophagic signaling, lipid droplet formation is impaired in FPLD2, as observed in an in vitro model of *LMNA*-lipodystrophy (Pellegrini et al., in preparation).

Aberrant differentiation or even determination of adipocyte precursors might play a major role in FPLD2 pathogenesis. Along this line, the Collas group showed that altered lamin A association with the RNA-binding protein Fragile X syndrome-related protein 1 (FXR1P) and upregulation of FXR1P in FPLD2 adipogenic precursors leads to conversion to the myogenic lineage [131]. However, a complex pathogenetic picture is emerging from FPLD2 studies, also involving the anti-adipogenic factors. For instance, the p.R482W mutation impairs differentiation-dependent lamin A binding to the MIR335 locus and overexpression of the MIR355 gene after adipogenic induction [25]. Moreover, tissue and depot specific effects might be related to lamin A tissue-specific interactions, such as the one with the adipocyte nuclear envelope protein TMEM120 [132].

Involvement of mTOR signaling in laminopathic adipose tissue defects has been also explored in *Lmna*^−/−^ mice [111] used to study cardiomyopathy and muscle dystrophy. In that mouse model, mTORC2 inactivation specifically in adipose tissue elicited weight gain and improved the whole body metabolism [111]. Importantly, high energy expenditure was observed in this mouse model, while rapamycin reversed this condition, indicating that mTOR-dependent signaling affected the rate of energy expenditure [121]. The reduction in adiposity in *Lmna*^−/−^ mice seems to be linked to lipolysis, which is decreased after rapamycin treatment. Very interestingly, mTOR was found to be aberrantly activated in adipose tissue, while rapamycin suppressed hyperactivated mTOR signaling, rescuing the phenotype. These results suggest a link between A-type lamin functions and mTOR signaling in adipose tissue and imply that not only adipose tissue homeostasis and differentiation, but also metabolic regulation may be related to altered mTOR regulation in the absence of a functional lamina (Figure 4) [121]. 

## 6. Progeroid Laminopathies

### 6.1. HGPS

Progeroid laminopathies include several forms linked to *LMNA* mutations and a few forms associated with *ZMPSTE24* gene variants or the *POLD1* gene [1]. Among *LMNA*-linked progeroid syndromes are HGPS, MADA, atypical-Werner syndrome, and atypical progeria syndrome [1]. MADB is linked to ZMPSTE24 mutations, while a form of mandibuloacral dysplasia also featuring hearing loss is associated with *POLD1* mutations [1]. 

HGPS is a rare genetic disorder characterized by very early onset accelerated ageing with hair loss, short stature, skin tightness, joint contractures, progressive cardiovascular disease resembling atherosclerosis, osteolysis of clavicles, mandible and terminal phalanges, osteoporosis, and death due to cardiovascular problems at an average age of 14.6 years [133]. Children with HGPS are healthy at birth but rapidly develop a progeroid phenotype [133]. HGPS is due to a sporadic mutation in the *LMNA* gene (c.1824C<T) [134,135], resulting in a silent polymorphism at codon 608 (G608G) that activates a cryptic splice site. This leads to a deletion of 50 amino acids near the C-terminus of prelamin A. The abnormal protein produced, called progerin, lacks the second site for endoproteolytic cleavage, and thus remains permanently farnesylated. Accumulation of progerin exerts toxic effects disrupting the integrity of nuclear envelope and leading to nuclear architecture abnormalities [136]. Even though G608G is the most frequent mutation in HGPS patients (at least 90% of all progeria cases), other mutations in the gene cause progeroid phenotypes classified as atypical progeria syndrome, with onset in the first decade, variable bone phenotype and lipodystrophy, or atypical-Werner syndrome, with onset of accelerated ageing in the second decade [137,138,139].

### 6.2. mTOR Signaling in HGPS

AKT-mTOR signaling has been analyzed in HGPS fibroblasts. AKT phosphorylation was reduced in HGPS cells [140], and mTOR phosphorylation, possibly leading to autophagy activation, was reduced in HGPS fibroblasts [141]. Moreover, in a mouse model of progeria, the Zmpste24 null mouse, which accumulates toxic levels of prelamin A, AKT, and S6 kinase phosphorylation were significantly reduced in liver and skeletal muscle, [140], suggesting activation of the autophagic signaling. Unexpectedly, in the same mouse model, genetic ablation of the prelamin A methyltransferase Icmt caused a significant activation of AKT and its downstream effectors in the mTOR pathway, leading to phosphorylation and degradation of p21 and reduced cellular senescence [140]. Thus, mTOR inactivation seems to play a dual role in progeroid cells, modulating both the autophagic signaling and p21-dependent cellular senescence [140]. Although autophagy is considered an anti-aging mechanism, increased levels of p21 are associated with senescence. Moreover, we did not observe any degradation of progerin in HGPS cells unless rapamycin was used to further inhibit the mTOR activity [141,142]. More recently, autophagy has been proposed as a mechanism to recycle nutrients in *Lmna* G609G progeroid mice [143], which were affected by severe weight loss and cachexia. In these mice subjected to high fat diet, LC3 II levels were reduced compared to individual under normal diet regimen [143]. The authors did not explore mTOR signaling but suggest that elevated energy intake by high fat diet could downregulate autophagy in progeroid mice. This complex picture needs further investigation, mostly to understand to which extent the HGPS phenotype could be improved by treatment with rapamycin or other rapalogs.

### 6.3. mTOR in Ageing Models

Several mouse models have been developed to better understand the effects of mTOR signaling in promoting aging and age-related phenotypes. Mice lacking S6K1 [144] and mice bearing heterozygous deletions of mTOR and mLST8 [145] show extended longevity. Moreover, mTOR hypomorphic mice display increased median lifespan, and they are healthier and seem to be protected from many age-related diseases [118]. 

Interestingly, many other studies support the effects of the mTOR signaling pathway on aging through a pharmacological approach [146,147,148,149,150]. Several evidences suggest that rapamycin could modulate a number of aging-related mechanisms and could be a potential anti-aging therapy, extending average and maximum lifespan in mice and delaying several age-related pathologies [148,151,152,153]. Rapamycin may also reverse features of ageing in mice, such as cardiac hypertrophy, liver degeneration, adrenal glands and endometrium decline, and tendon elasticity [137]. 

Besides to limit the set of problems caused by prolonged exposure to rapalogs, different approaches have been carried out, reporting that an intermittent rapamycin treatment schedule is associated with fewer side effects on the immune system and on glucose metabolism, and with a lifespan extension in mouse models [154,155,156]. Another possible dosing regimen for delaying the aging phenotype and minimizing the side effects has been suggested by Mannick and colleagues, proposing low-dose and short-time everolimus administration [157]. Finally, a very recent paper has demonstrated that a methionine restriction diet extended lifespan in *LmnaG609G/G609G* and *Zmpste24*−/− HGPS mouse models, rescuing the pathologic phenotype, by reversing the transcriptome alterations in inflammation and DNA-damage response genes, improving the lipid profile and changing bile acid levels and conjugation. Methionine restriction also induced downregulation of the mTOR pathway, suggesting the existence of a metabolic signaling involved in the longevity extension achieved by the methionine restriction diet [158].

### 6.4. mTOR Inhibitors in HGPS

Rapamycin reduces progerin levels in HGPS cells, avoids farnesylated prelamin A accumulation, and rescues physiological chromatin dynamics [142]. Temsirolimus, another rapalog, has been tested in HGPS cells [68]. Nevertheless, mitochondrial dysfunction [159] and elevated DNA damage observed in HGPS cells are not rescued by the drug [68]. Combination of drugs is currently considered the most promising approach to HGPS therapy, not only because of the complex clinical condition but also to take advantage of the synergistic effects of drugs that allow for the use of a low dosage, thus limiting toxicity. In a study we performed in HGPS cells, all-trans retinoid acid (ATRA) and rapamycin were shown to synergistically improve the lamin A to progerin ratio. The beneficial effect leading to reduction of DNA damage and improvement of BAF and chromatin dynamics was elicited through a transcriptional effect, possibly due to ATRA, and through rapamycin-dependent activation of progerin autophagic degradation (Figure 4) [160]. The combined treatment is currently being tested in animal models. The administration of rapalogs with the anti-diabetes drug metformin, able to minimize adverse effects, may represent a further strategy for the use of mTOR inhibitors [160]. Metformin is a regulator of the mTORC1-dependent translation process, activating the AMP-activated protein kinase (AMPK) axis [161] and directly inhibiting mTOR [162]. This drug shows anti-aging effects in many models [163], and an observational and retrospective study in diabetic patients revealed that metformin treatment leads to a strong decrease in all-cause death and in the onset of age-related diseases [164]. Recently, it has reported in a mouse model that a combination of rapamycin and metformin reduces the strong metabolic deficits caused by rapamycin treatment [120]. Interestingly, in HGPS fibroblasts and *Lmna* G609G/G609G mouse fibroblasts, metformin diminishes progerin expression [165], suggesting a possible therapeutic potential of metformin for HGPS. 

A recent approach to HGPS treatment involves lonafarnib (a prelamin A and progerin farnesylation inhibitor [166] combined with everolimus (an analogue of rapamycin that has a more favorable pharmacokinetic profile) and an ongoing phase I/II trial has been launched by the Progeria Research Foundation [NCT02579044]. 

Another strategy to modulate autophagy and decrease the progerin levels in HGPS patients’ cells employed proteasome inhibitor MG132 as an activator of lysosomal degradation in response to proteasome inhibition. Moreover, progerin degradation, following MG132 treatment, was observed in HGPS IPSC-derived cell lines as well as in vivo in an *Lmna* G609G/G609G mouse model, showing an amelioration of cellular HGPS phenotype (Figure 4) [167]. Finally, various nanoparticles (NPs) have demonstrated an ability to modulate mTOR activity and proliferation. This approach to mTOR modulation warrants further investigation [168,169].

### 6.5. MADA and MADB

MAD is a rare laminopathy characterized by progeroid features, including growth retardation, fat distribution, and metabolic abnormalities (diabetes, glucose intolerance, and insulin resistance) and severe osteolysis and osteoporosis [1,141]. MADA fibroblasts show nuclear blebbing and reduced proliferation. Patients with MADA have a less severe phenotype as compared to MADB harboring *ZMPSTE24* mutations, consistent with the accumulation of a higher amount of prelamin A in MADB. Several therapeutic approaches have been reported to rescue the cellular abnormalities, such as farnesyl transferase inhibitors, statins, and bisphosphonate [16].

Interestingly, some activation of autophagy in MADA has been suggested by studies performed with chloroquine. In MADA fibroblasts subjected to chloroquine treatment, prelamin A level is increased, while, as expected, the autophagic process is impaired (Figure 3) [170]. Thus, it appears that an autophagic mechanism of removal of mutated prelamin A (MADA cells carry homozygous *LMNA* mutations and are devoid of wild-type lamin A/C) is activated in those cells [170]. Consistent with this observation, inhibition of mTOR by rapamycin triggers lysosomal degradation of farnesylated prelamin A in MADA fibroblasts and rescues markers of cellular senescence as well as chromatin epigenetic mechanisms [5,170]. In contrast to what observed in MADA fibroblasts, osteoblast-like cells overexpressing lamin A R527H showed activation of the mTOR pathway, although mTOR itself was not phosphorylated. Inhibition of this pathway by everolimus treatment significantly improved the mutant phenotype and its pathogenetic pathways, including the ability of R527H *LMNA* osteoblasts to stimulate osteoclastogenesis, suggesting that everolimus can be explored as a therapeutic approach for MADA [32]. 

Rapamycin treatment may be a therapeutic strategy for MADB as well. Indeed, it has been demonstrated that inhibition of the mTOR signaling pathway induces improvement of nuclear aberrations and ameliorates the overall phenotype of fibroblasts from MADB patients, even if changes in the phosphorylation status of mTOR have not been determined (Figure 4) [171].

## 7. Perspectives

From a basic point of view, the main finding of mTOR studies conducted in models of laminopathies is that functional lamin A/C is required for mTOR-dependent pathways that regulate autophagy and fibrogenic processes. The link between a functional lamina and autophagy is consistent with the observed involvement of autophagy in lamina homeostasis through degradation of defective or excess A or B type lamins [142,160,170,172]. Thus, a feedback mechanism could be hypothesized, whereby proteins of the nuclear lamina inhibit mTOR activity and trigger autophagy to maintain their physiological levels. This hypothesis has been widely proven for lamin B [172]. Mutated lamins, including the R527H-mutated lamin A/C and prelamin A found in many cases of MADA and progerin, likely fail to trigger efficient mTOR-dependent autophagic signaling, thus causing protein accumulation. However, while EDMD2 and FPLD2 appear to be characterized by activation of the AKT/mTOR pathway either through mTOR itself or through direct phosphorylation of p70S6 kinase, ultimately impairing the autophagic activity [30,32,111,121,173], HGPS and MADA cells show some activation of the autophagic pathway. Nevertheless, despite this relevant difference in mTOR signaling, in experimental models of all these laminopathies, rapamycin, everolimus, and temsirolimus have been demonstrated to efficiently degrade toxic molecules and/or rescue the phenotype. Despite the known side effects of rapamycin and rapalogs, those results suggest that therapeutic approaches based on mTOR inhibition and activation of autophagy be explored. However, recent findings showing that other mTOR dependent mechanisms such as nutrient intake and energy expenditure are affected in laminopathies suggest a more complex therapeutic strategy aimed at rescuing good metabolic conditions while reducing levels of mutated lamins.

## Figures and Tables

**Table 1:** The most representative laminopathies are listed. * nomenclature for these forms is provisional; ** potential hotspot; *** cardiomyopathy with conduction defect type 1A (DCM1A).

Disease	Gene	Protein	Hotspot	Inheritance	Phenotype	Ref.
*Muscular Laminopathies*						
EDMD2	*LMNA*	Lamin A/C	R453	AD	Joint contractures, muscle weakness and wasting, cardiomyopathy	[39,40]
EDMD1	*EMD*	Emerin		X-linked	Joint contractures, muscle weakness and wasting, cardiomyopathy	[39,40]
EDMD3	*LMNA*	Lamin A/C		AR	Joint contractures, muscle weakness and wasting, cardiomyopathy	[39,40]
EDMD4	*SYNE1*	Nesprin 1		AD	Joint contractures, muscle weakness and wasting, cardiomyopathy	[39,41]
EDMD5	*SYNE2*	Nesprin 2		AD	Joint contractures, muscle weakness and wasting, cardiomyopathy	[39,41]
EDMD6	*FHL1*	FHL1		X-linked	Joint contractures, muscle weakness and wasting, cardiomyopathy, vocal cord involvement	[40,42]
EDMD7	*TMEM43*	LUMA		AD	Muscle weakness, cardiomyopathy with cardiac conduction defects	[40,43]
LGMD1B	*LMNA*	Lamin A/C		AD	Joint contractures, muscle weakness and wasting, cardiomyopathy	[39,40]
L-CMD	*LMNA*	Lamin A/C		AD	Severe and early onset muscle weakness and wasting, contractures, delayed/absent motor milestones, dropped head, cardiomyopathy	[39,40]
MD*	*SUN1, SUN2*	SUN1, SUN2		AD	Cardiomyopathy, skeletal muscle weakness and wasting	[37,44]
*Cardiomyopathies*						
DCM1A***	*LMNA*	Lamin A/C		AD	Dilated cardiomyopathy and conduction defects	[35,45]
DCM	*SYNE1*	Nesprin 1		AD	Dilated cardiomyopathy	[46,47]
DCM-CD	*TMPO*	Lap2 α			Dilated cardiomyopathy joint contractures	[48,49]
*Lipodystrophies*						
FPLD2	*LMNA*	Lamin A/C	R482	AD	Loss of subcutaneous fat, accumulation of fat in the neck, diabetes, polycystic ovary syndrome	[50,51]
APL	*LMNB2*	Lamin B2		AR	Symmetrical loss of subcutaneous fat from the face, neck, upper extremities, thorax, and abdomen, sparing the lower extremities	[52,53]
*Progeroid Laminopathies*						
HGPS	*LMNA*	Lamin A/C	G608		Premature and accelerated aging, growth arrest, lipodystrophy mandible, clavicle, phalanges osteolysis, osteoporosis, atherosclerosis	[54,55]
APS	*LMNA*	Lamin A/C			Premature aging, lipodystrophy, cardiovascular disease, short stature, diabetes and alopecia	[24,56]
A-WS	*LMNA*	Lamin A/C		AD	Late onset premature aging, atherosclerosis, lipodystrophy, diabetes	[1,57]
MADA	*LMNA*	Lamin A/C	R527	AR AD	Mandible, clavicle, phalanges osteolysis, osteoporosis, partial lipodystrophy, short stature, metabolic abnormalities, mildly accelerated aging	[1,58]
MADB	*ZMPSTE24*	ZMPSTE24	1085dupT **	AR AD	Accelerated aging, mandible, clavicle, phalanges osteolysis, osteoporosis, generalized lipodystrophy, short stature, metabolic abnormalities	[1,59]

## Abbreviations

4E-BP1	Eukariotic translation initiation factor 4E-binding protein 1
AMPK	AMP-activated protein kinase
ATG14L	ATG autophagy related-14 like
ATM	Atacsia telangectasia-mutated
ATR	Atacsia telangectasia- and RAD3-related
ATRA	All-trans retinoid acid
BAF	Barrier to autointegration factor
CMT2B1	Charcot–Marie tooth neuropathy
DEPTOR	DEP domain-containing mTOR-interacting protein
DNA-PK	DNA-dependent protein kinase
EDMD	Emery-Dreifuss muscular dystrophy
eEF2K	Eukaryotic elongation factor 2 kinase
eIF-4E	Eukaryotic initiation factor-4E
FAT	FKBP/ATM/TRRAP
FATC	FRAP/ATM/TRRAP/carboxy terminal
FKBP-12	FK506-binding protein-12
FoxO	Forkhead box O
FPLD2	Familial partial lipodistrphy 2
FRB	FKBP, rapamycin-binding
FXR1P	Fragile X syndrome-related protein 1
GAP	PTPase activating protein
HEAT	Huntingtin/elongation factor 3/A subunit of protein phosphatase-2A/TOR1
HGPS	Hutchinson–Gilford progeria syndrome
HHS	“Heart hand” syndrome
HIF1 alpha	Hypoxia inducible factor 1
hSMG-1	Human suppressor of morphogenesis in genitalia-1
IGFR	Insulin-like growth factor receptor
IR	Insulin receptor
IRS1	Insulin receptor substrate 1
LAD	Lamina-associated domain
LC3	Microtubule-associated protein 1 light chain 3
L-CMD	Cardiomyopathy-lmna-linked congenital muscular distrophy
LKB1	Liver kinase 1
MADA	Mandibuloacral dysplasia
mLST8	Mammalian sethal with SEC13 protein 8
MRTFA	Mechanoresponsive myocardin-related transcription factor A
mSin	Mammalian stress-activated protein kinase Interacting protein 1
mTOR	Mechanistic target of rapamycin
PI3K	Phosphoinositide 3-kinase
PIKK	Phosphoinositide kinase-related family of protein kinases
PKC	Protein kinase C
PPARgamma	Peroxisome proliferatior-activated receptor gamma
PRAS40	Proline-rich AKT substrate 1 40 kDa
PROTOR 1/2	Protein observed with Rictor
RAPTOR	Regulatory-associated protein of TOR
RD	Restrictive dermopathy
RHEB	Ras homolog enriched in brain
RICTOR	Rapamycin-insensitive companion of TOR
RPS6	Ribosomal protein S6
RTK	Receptor tyrosine kinase
S6K1	P70 ribosomal S6 kinase 1
SGK1	Serum and glucocorticoid-activated kinase 1
SREBP1	Sterol regulatory element-binding protein 1
TSC2	Tuberous sclerosis complex 2
UCP1	Uncoupling protein 1
UPS	Ubiquitin proteasome system
Vps34	Vacuolar protein sorting 34
